# Lung ultrasound training: how short is too short? observational study on the effects of a focused theoretical training for novice learners

**DOI:** 10.1186/s12909-024-05148-0

**Published:** 2024-02-21

**Authors:** Silvia Mongodi, Raffaella Arioli, Attilio Quaini, Giuseppina Grugnetti, Anna Maria Grugnetti, Francesco Mojoli

**Affiliations:** 1 Anesthesia and Intensive Care, Fondazione IRCCS Policlinico S. Matteo, Rianimazione I, Viale Golgi 19, 27100 Pavia, Italy; 2 Department of Health Professions, Fondazione IRCCS Policlinico S. Matteo, Pavia, Italy; 3 Department of Clinical-Surgical, Diagnostic and Pediatric Sciences, Unit of Anesthesia and Intensive Care , University of Pavia, Pavia, Italy

**Keywords:** Lung ultrasound, Nurse teaching, Nurse training, Point-of-care ultrasound, LUS

## Abstract

**Background:**

Lung ultrasound has been increasingly used in the last years for the assessment of patients with respiratory diseases; it is considered a simple technique, now spreading from physicians to other healthcare professionals as nurses and physiotherapists, as well as to medical students. These providers may require a different training to acquire lung ultrasound skills, since they are expected to have no previous experience with ultrasound. The aim of the study was to assess the impact of a short theoretical training focused on lung ultrasound pattern recognition in a population of novice nurse learners with no previous experience with ultrasound.

**Methods:**

We included the nurses attending a critical care advanced course for nurses performed at the University of Pavia. Images’ interpretation skills were tested on two slide sets (a 25-clip set focused on B-pattern recognition and a 25-clip set focused on identification of pleural movement as lung sliding, lung pulse, lung point, no movement) before and after three 30-minute teaching modules dedicated to general ultrasound principles, B-lines assessment and lung sliding assessment. A cut off of 80% was considered acceptable for correctly interpreted images after this basic course.

**Results:**

22 nurses were enrolled (age 26.0 [24.0–28.0] years; men 4 (18%)); one nurse had previous experience with other ultrasound techniques, none of them had previous experience with lung ultrasound. After the training, the number of correctly interpreted clips improved from 3.5 [0.0–13.0] to 22.0 [19.0–23.0] (*p* < 0.0001) for B-pattern and from 0.5 [0.0–2.0] to 8.5 [6.0–12.0] (*p* < 0.0001) for lung sliding assessment. The number of correct answers for B-pattern recognition was significantly higher than for lung sliding assessment, both before (3.5 [0.0–13.0] vs. 0.5 [0.0–2.0]; *p* = 0.0036) and after (22.0 [19.0–23.0] vs. 8.5 [6.0–12.0]; *p* < 0.0001) the training. After the training, nurses were able to correctly recognize the presence or the absence of a B-pattern in 84.2 ± 10.3% of cases; lung sliding was correctly assessed in 37.1 ± 15.3% of cases.

**Conclusions:**

Lung ultrasound is considered a simple technique; while a short, focused training significantly improves B-pattern recognition, lung sliding assessment may require a longer training for novice learners.

**Trial registration:**

Not applicable.

## Background

**Fig. 1:**
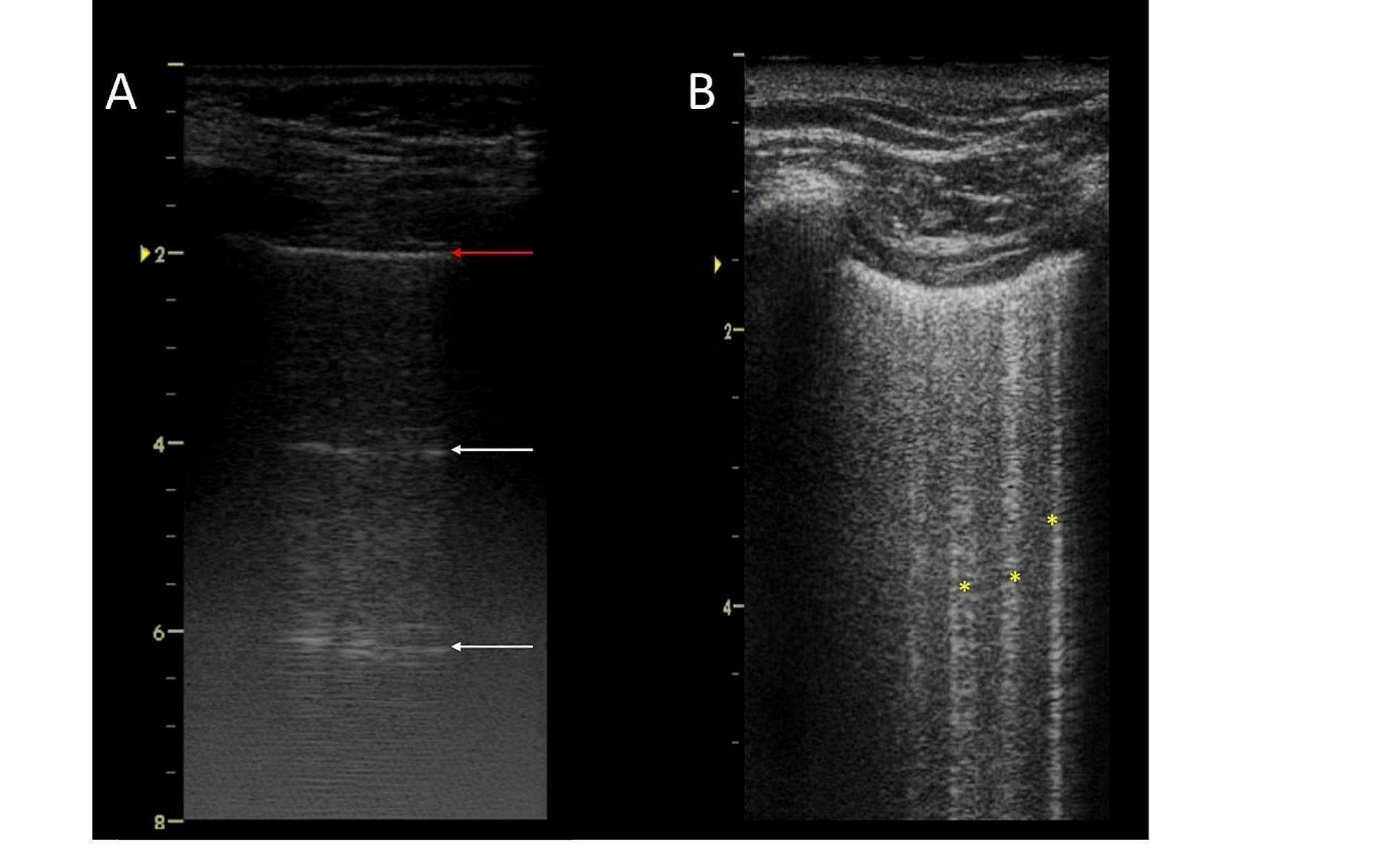
Longitudinal scans of an intercostal space. (**A**) The pleural line (red arrow) is visualized between the ribs; reverberation artifacts beneath the pleura (A-lines– white arrows) indicate normal aeration and rule out cardiogenic edema. (**B**) 3 B-lines are visualized: vertical artifacts deriving from the pleura, reaching the bottom of the screen while erasing the A-lines: this B-pattern supports the diagnosis of a cardiogenic edema as the cause of acute dyspnea

Lung ultrasound (LUS) spread in the last years for the assessment of patients with respiratory diseases [1–7]. The recognition of simple patterns allows easily distinguishing the main causes of acute respiratory distress. While a normal lung presents horizontal reverberation artifacts called A-lines (Fig. 1A) [2], an increase in lung density [5, 6] generates vertical hyperechoic artifacts called B-lines; they become significant when ≥ 3 in an intercostal space, defining a B-pattern (Fig. 1B) [9–11]. In a patient with wheezing, the absence of B-pattern rules out cardiogenic edema with 100% sensitivity, orienting to obstructive diseases; on the contrary, the B-pattern identifies cardiogenic edema with 92% specificity [12]. The interest of this application has also been demonstrated in extra-hospital medicine [13–15]. In the context of a trauma patient, pneumothorax is easily ruled out by the lung sliding, corresponding to the visceral pleura sliding against the parietal one [7, 16] or the lung pulse [17]. If no pleural movement is visualized, pneumothorax can be confirmed with 100% specificity by the lung point, where visceral and parietal pleura regain contact [18, 19].

Being an inexpensive, non-irradiating and informative bedside tool, the interest in acquiring ultrasound skills recently expanded to allied healthcare professionals, as physiotherapists [20], nurses [21–25] and critical care paramedics [26], as well as to medical students.

In critical care nurses’ daily activity, LUS may be of help in assessing acute patients [27–28], for example in grading the severity of the disease (triage) in Emergency Department. In such scenarios, nurses usually use auscultation for thoracic evaluation, although it has a lower diagnostic accuracy than LUS [3, 29] and an unclear training pathway. Mumoli et al. described how the nurse-performed LUS improved early diagnosis of dyspnea caused by cardiogenic pulmonary edema [22]. A nurse-performed ultrasound integrated approach has been also suggested to improve the management of new-coronavirus disease (COVID-19) critically ill patients [30].

To correctly use LUS, nurses should receive an adequate training [23] and the correct path to be followed is still unclear. A short LUS training showed to significantly improve image recognition skills in emergency physician [31]; however, knowledge and use of ultrasound in generally higher in medical staff, potentially requiring a shorter training.

The aim of the present study was to assess the efficacy of a short theoretical training in a population of ultrasound-novice nurses in reaching adequate LUS pattern recognition.

## Methods

We selected a population with expected limited or no LUS knowledge: in a critical care advanced course for nurses performed at the University of Pavia (“Master di I livello di Infermiere in Area Critica”, an equivalent of Advanced Practice Registered Nurses in North America), students underwent a LUS theoretical training and were tested before and after the training to assess the improvement in their image recognition skills of two different LUS patterns (B-lines and lung sliding). The need for approval was waived by the ethic committee (Comitato Etico Referente per l’Area di Pavia) since it’s a training protocol and no patients are involved. The director of the university course involved in the training protocol approved the protocol (agreed that we performed the protocol on his students); all participants signed an informed consent.

Before the training, nurse trainees were asked to complete a pre-test evaluation consisting in the interpretation of two sets of clips, 25 each: the first focused on the recognition of presence/absence of a B-pattern, the second focused on lung sliding and the recognition of one of the following patterns: lung sliding, lung pulse, lung point, no movement. A score was attributed to each student on the basis of the number of correct answers (1 correct answer = 1 point; score ranging from 0 to 25 in each set).

The nurses were adequately positioned in a classroom, respecting a distance among the trainees to avoid any kind of bias related to a possible collaboration among them. The pre-test questionnaire was not corrected immediately, and nurses were not informed about correct or incorrect answers, so as not to create a knowledge bias.

Trainees then underwent three consecutive training modules: general principles of ultrasound (30 min); B-lines assessment (30 min) and lung sliding assessment (30 min). Lectures were given by a physician expert in thoracic ultrasound practice and teaching (SM). The clips visualized during the training modules were different from those used in the pre/post-test. After the training, students were asked to complete the post-test evaluation, identical to the pre-test.

At the end of the data collection, pre- and post-test were corrected.

General information about students were also collected (age, sex, length of work experience, previous experience in critical care (i.e., previous work in intensive care unit, emergency department, pre-hospital medicine), previous experience and training in general ultrasound, previous experience and training in LUS, frequency of LUS use by physicians in the student’s unit/ward, availability of an ultrasound machine in the student’s unit/ward.

### Statistical analysis

A sample size of 14 students (11 + 30% drop out) was required to detect a mean score modification of 6.9 ± 4 points in each slot, with alpha error 0.01 and power 0.99. Data for sample size computation were derived by Noble et al. [30] and computed with G-Power. P-value ≤ 0.01 was considered significant (two-sided). A cut off of 80% was considered acceptable for correctly interpreted images after a basic course for novices.

Median and interquartile range [IQR] were used for the quantitative variables, and number and percentages for the categorical ones. Normal distribution was assessed by Shapiro-Wilk test; non-parametric tests were preferred in consideration of the limited sample size. Comparison between pre- and post-test scores were performed by Wilcoxon/Mann-Whitney U-test for paired data, therefore using participants as their own control.

All the analyses were conducted with STATA/SE for Macintosh, version 14.2.

## Results

23 nurse trainees attending the class were enrolled in the training program; one was excluded from final analysis since he joined the class too late to perform the pre-test.

The population characteristics of 22 nurses are displayed in Table 1. Nurses were mainly young women at the beginning of their professional experience. While interested in critical care, most of them worked in non-critical care units/wards. An ultrasound machine was available in their units/wards in 72.7% and was used by physicians to perform LUS at least occasionally in 68.2%. However, only one trainee had had previous training in general ultrasound and none in LUS, reaching therefore the target of a trainees’ population with no previous knowledge in LUS.

**Table 1:** Characteristics of the population of nurse trainees involved in the lung ultrasound training program

Nurse trainee’s population (*n* = 22)	
Age– years (median, [IQR])	26.0 [24.0–28.0]
Male sex– n (%)	4 (18)
Work experience– years (median, [IQR])	2.0 [1.0–4.0]
Activities in critical care fields– n (%)	
• Intensive Care Unit • Emergency Department • Pre-hospital medicine • Others (respiratory, cardiac intensive care) • None	3 (13.6) 1 (4.6) 0 (0.0) 4 (18.2) 14 (63.6)
Ultrasound machine availability in trainee’s unit/ward– n (%)	
• Available and dedicated to the unit • Available but shared with another unit/ward • Not available	14 (63.6) 2 (9.1) 6 (27.3)
Previous trainee’s use of ultrasound– n (%)	
• Systematic • Occasional • No use	1 (4.5) 8 (36.4) 13 (59.1)
Previous trainee's training in ultrasound - n (%)	
• Yes • No	1 (4.5) 21 (95.5)
Previous trainee’s training in lung ultrasound– n (%)	
• Yes • No	0 (0.0) 22 (100)
Use of lung ultrasound in trainee’s unit/ward– n (%):	
1. By doctors:	
• Frequent • Occasional • No use	7 (31.8) 8 (36.4) 7 (31.8)
2. By nurses:	
• Frequent • Occasional • No use	0 (0.0) 2 (9.1) 20 (90.9)

IQR: inter-quartile range

Before the training, median scores were 3.5 [0.0–13.0] points out of 25 for B-pattern recognition and 0.5 [0.0–2.0] points out of 25 for lung sliding assessment; in both cases, trainees were mostly unable to answer the questions, as expected (Table 2).

**Table 2:** Impact of the focused training on B-pattern and Lung sliding interpretation

		Pre-test On 25 clips	Post-test On 25 clips	p*
**B-Pattern**	**Correct** Number– median [IQR]	3.5 [0.0–13.0]	22.0 [19.0–23.0]	< 0.0001
	**Wrong** Number– median [IQR]	3.5 [0.0–7.0]	2.0 [1.0–3.0]	0.1000
	**Missing** Number– median [IQR]	16.0 [3.0–25.0]	0.5 [0.0–4.0]	< 0.0001
**Sliding**	**Correct** Number– median [IQR]	0.5 [0.0–2.0]	8.5 [6.0–12.0]	< 0.0001
	**Wrong** Number– median [IQR]	1.0 [0.0–6.0]	10.0 [7.0–13.0]	0.0002
	**Missing** Number– median [IQR]	23.0 [18.0–25.0]	4.0 [2.0–9.0]	< 0.0001

Median number of correct, wrong and missing answers before and after theoretical training in identification of lung sliding / lung pulse / lung point and B-pattern. *Wilcoxon signed rank test for paired data

After the training, median scores significantly improved to 22.0 [19.0–23.0] (*p* < 0.0001) points for B-pattern recognition and to 8.5 [6.0–12.0] (*p* < 0.0001) points for lung sliding assessment. In both cases, the median number of missing answers significantly decreased (B-pattern recognition: from 16.0 [3.0–25.0] to 0.5 [0.0–4.0], *p* < 0.0001; lung sliding assessment: from 23.0 [18.0–25.0] to 4.0 [2.0–9.0], *p* < 0.0001) (Table 2).

The number of correct answers for B-pattern recognition was significantly higher than for lung sliding assessment, both before (3.5 [0.0–13.0] vs. 0.5 [0.0–2.0]; *p* = 0.0036) and after (22.0 [19.0–23.0] vs. 8.5 [6.0–12.0]; *p* < 0.0001) the training.

After the training, nurses were able to correctly recognize the presence or the absence of a B-pattern in 84.2 ± 10.3% of cases; lung sliding was correctly assessed in 37.1 ± 15.3% of cases.

## Discussion

The main finding of this study is that a short, focused training in ultrasound-novice nurses allows reaching a satisfactory percentage of correctly interpreted clips for B-pattern recognition but not for lung sliding assessment.

A nurse-performed ultrasound assessment has been suggested to improve nurses’ activities, in particular in pre-hospital setting and in Emergency Department, to integrate their primary evaluation and to improve patient’s triage and management. For example, in the recent coronavirus outbreak, a focused ultrasound assessment showed to improve the triage of acutely ill patients in Emergency Department [31], while reducing the exposure of additional healthcare professional [32]. However, so far, there was only one study focused on nurses training in basic LUS [22].

For physicians, thoracic ultrasound has been demonstrated to be easy to learn for simple applications as B-pattern and lung sliding recognition [30]; a short training provides adequate skills for basic knowledges, in particular for basic images' interpretation. However, physicians may more easily have a previous experience with ultrasound machines and techniques.

The number of correct answers was extremely low before the training; this may be explained by the lack of previous training and experience in both general and lung ultrasound. Only one nurse had had in fact previous training in ultrasound. Our nurses were novices with mostly no ultrasound exposure, which may explain the high number of missing answers in the pre-test.

After the training, the percentage of correctly interpreted images significantly improved. For B-pattern recognition it reached 84.2%; a comparable result was obtained after a similar training for emergency physicians [30]. Lung sliding resulted to be more difficult to assess, both before and after the training. The percentage of correct interpretation after the training significantly improved but only reached 37.1%. This result is lower than what obtained after a similar training in emergency physicians and may also be explained by the extremely limited previous experience in lung ultrasound in the studied population of nurses. Such a low percentage limits the possible application of lung ultrasound for pneumothorax detection after a short training; however, these results suggest that a longer and/or repeated training for unexperienced providers may improve the results on images recognition skills and could be a starting point for new perspectives concerning lung ultrasound application among healthcare providers with no previous experience with ultrasound as nurses, physiotherapists or medical students.

The difference between the improvement in pattern recognition of B-lines and lung sliding may have multiple explanations. First, the test focused on B-pattern recognition implied yes or no questions, while the test focused on pleural movements required to distinguish four patterns (lung sliding, lung pulse, lung point, absence of movement), thus more information had to be retained from the short training. Second, the teaching methods and the instructional style were kept as similar as possible for B-pattern and lung sliding assessment; we decided not to introduce the M-mode in a basic course, but we know this is a recommended instrument to better distinguish the pleural movements, since its higher frame rate increases its accuracy [1, 2]. Third, limited data are available on the specific training time required for B-pattern and lung sliding recognition respectively, but we could speculate an intrinsic difference in the identification of the two patterns: in fact, the first automated systems for the identification of the B-lines were developed more than 10 years ago [34] while deep learning and artificial intelligence were required to recently obtain a reliable automatic recognition of lung sliding [35]. Moreover, while the expert’s eye remains the gold standard for the interpretation and the quantification of the B-lines [36], the quantification of the lung sliding is more challenging and may require advanced tools as the speckle tracking [37].

This study presents many limitations: first, the small number of subjects limits the generalizability of the findings. Second, it only focused on images’ recognition skills; the training required for image acquisition was not tested and should be explored in further studies. Moreover, we didn’t explore if images’ recognition skills persisted at different time intervals (e.g., after 1 day, 1 week, 1 month). A limited number of basic signs were tested (B-lines and pleural movements), thus excluding other significant lung ultrasound findings as consolidations [38] or air-bronchogram within consolidations [39] that help in the diagnosis of pneumonia in the critically ill. Finally, a reference standard for the test was not available (i.e., the total score reported by a sample of experts in the field); however, the clips used for the tests were good quality images, with findings that are considered basic [40], so the percentage of correct answers by a pool of experts was expected to be near to 100%.

## Conclusions

LUS is considered a simple technique; while a short, focused training significantly improves B-pattern recognition, lung sliding assessment may require a longer training for novice learners.

## Data Availability

The datasets used and/or analysed during the current study are available from the corresponding author on reasonable request.

## Abbreviations

LUS: Lung Ultrasound
